# Primary Cutaneous Histoplasmosis in a HIV-Positive Individual

**DOI:** 10.4103/0974-777X.62884

**Published:** 2010

**Authors:** Biju Vasudevan, Bahal Ashish, Sagar Amitabh, Mohanty A P

**Affiliations:** *Department of Dermatology, MH Shillong, Shillong, Meghalaya, India*

**Keywords:** Histoplasmosis, Fungus, Human immunodeficiency virus, Itraconazole

## Abstract

A 31-year-old human immunodeficiency virus-positive male who presented to the Dermatology Outpatient Department with complaints of red, raised lesions on the face of 2 weeks duration was, on examination, found to have multiple papulonodular lesions on the face with associated cervical and axillary lymphadenopathy. There was history of local injury on the face 6 months prior to the development of symptoms. Skin biopsy revealed multiple round to oval spores with surrounding halo intracellularly, confirming the diagnosis of cutaneous histoplasmosis. No systemic involvement was detected on further investigations. The patient responded to oral antifungals in a short duration, confirming the local nature of the presentation. This is probably the first time in the literature that a primary cutaneous manifestation of histoplasmosis is being described in an immunocompromised individual.

## CASE HISTORY

A 31-year-old male, resident of Manipur, India and known to be human immunodeficiency virus (HIV)-positive since the last 4 months (acquired through heterosexual contact 4 years back) presented to the Skin outpatient clinic with complaints of red raised, itchy and oozing lesions over the face of 2 weeks duration. Preceding history of excessive sun exposure was present. He had an exactly similar episode about 4 months back, which was diagnosed as photoallergic dermatitis and regressed in a period of 2 weeks during which period he was also on antiretroviral therapy and tablet fluconazole 150 mg daily. The patient had temporarily discontinued all medications voluntarily 2 weeks before the occurrence of the second episode. He gave history of working at farms for the last 15 years and also a splinter injury on his forehead 6 months back.

**Figure 1:**
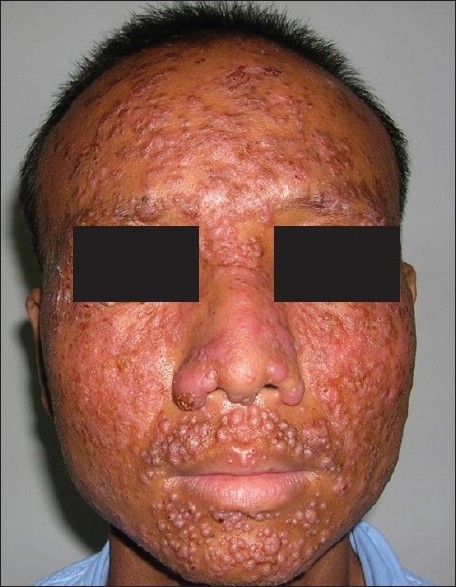
Nodules and plaques on the face with few pearly-white lesions around the eyes

Examination revealed significant axillary and cervical lymphadenopathy. Systemic examination was unremarkable. Dermatological examination revealed multiple skin-colored to erythematous papules and nodules over the middle and lower part of the face and crusted papules with plaques on the forehead and malar areas [Figure 1]. The lesions were mildly scaly and nontender with mild serous discharge. Few umbilicated pearly white lesions were present on the eyelids and perioral regions.

**Figure 2:**
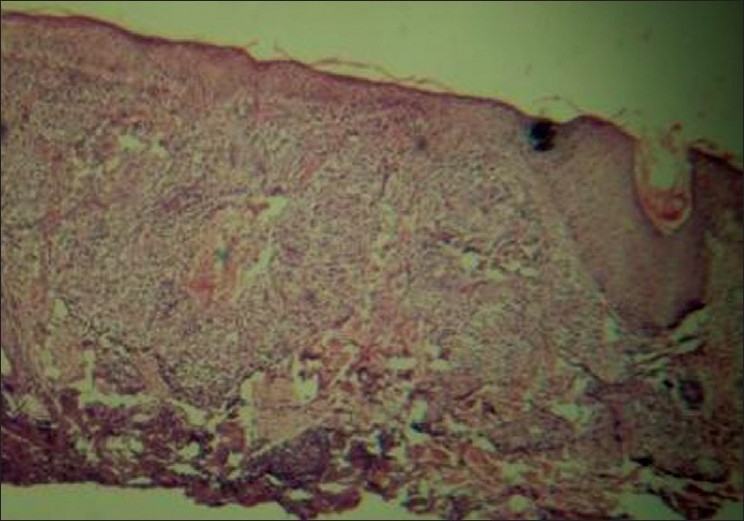
Skin biopsy showing thinned-out epidermis with multiple dermal granulomas comprising of lymphocytes, histiocytes and plasma cells (Hematoxylin and eosin stain–10×)

**Figure 3:**
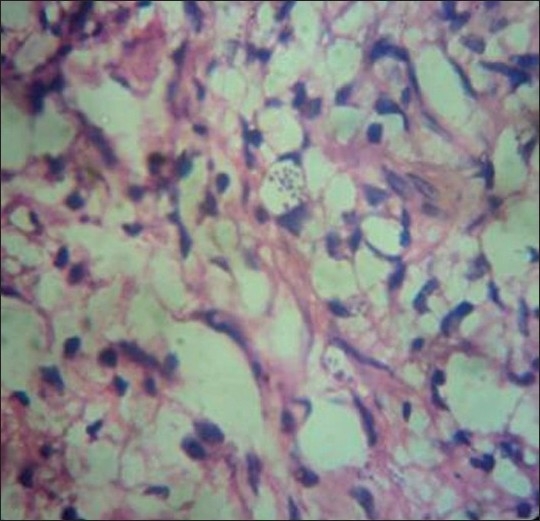
Higher magnification showing clusters of round fungal spores intracellularly (Hematoxylin and eosin stain–40×)

**Figure 4:**
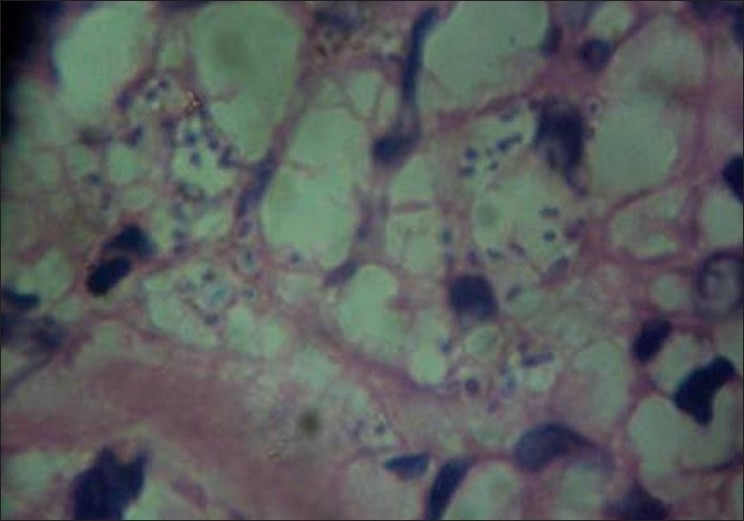
Oil immersion photomicrograph depicting halos around the intracellular spores (Hematoxylin and eosin stain–100×)

**Figure 5:**
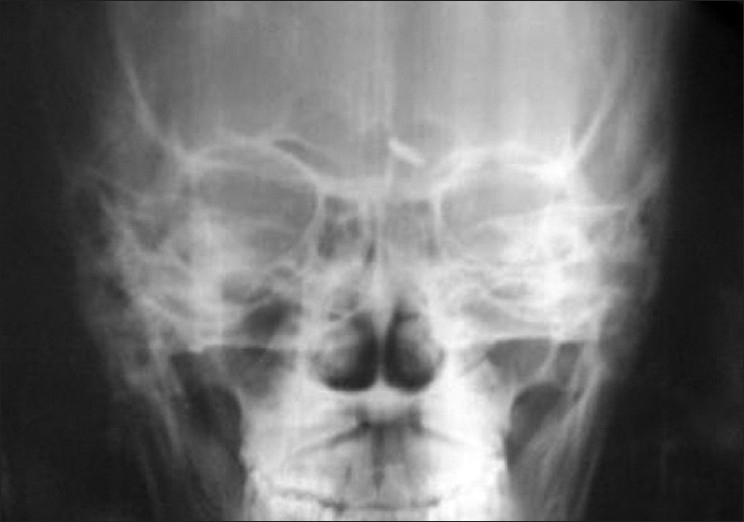
X-ray of the skull showing persisting splinter in the frontal area

**Figure 6:**
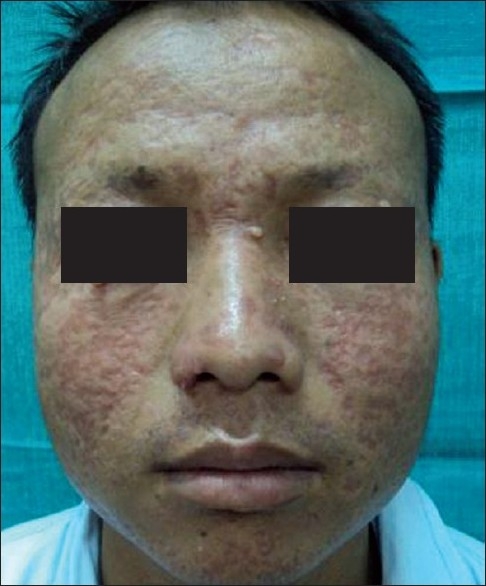
Complete regression of histoplasmosis lesions after treatment, with persisting molluscum lesions

Investigations revealed hemoglobin of 9 gm/dl with hypochromic microcytic peripheral blood smear. Other hematological and biochemical investigations were within normal limits. Skin biopsy from the lesions on hematoxylin and eosin stain revealed a thinned-out epidermis and dense collections of foamy macrophages occupying both papillary and reticular dermis. Few interspersed lymphocytes and plasma cells were also found [Figure 2]. Higher magnification showed the macrophages as having multiple intracellular fungal spores of size 2–4 μm [Figure 3]. Oil immersion microscopy identified clear surrounding haloes around all the spores [Figure 4]. Culture on Sabourad's agar showed fluffy white colonies. Thus, a diagnosis of cutaneous histoplasmosis was arrived at. Fine needle aspiration cytology (FNAC) from the lymph nodes showed nonspecific inflammation, and stains for fungal spores and acid fast bacilli were negative. X-ray of the skull showed the splinter to be persisting [Figure 5] while X-ray and contrast enhanced computed tomography (CECT) scan of the chest were normal. Further investigations revealed no involvement of any other organ, including adrenals, bones and central nervous system. CD4 count was found to be 21 cells/μl. The patient was treated with oral Itraconazole 200 mg twice daily and the cutaneous lesions regressed in 4 weeks [Figure 6]. The molluscum contagiosum lesions however persisted.

## DISCUSSION

Histoplasmosis is an opportunistic fungal infection caused by inhalation of dimorphic fungus *Histoplasma capsulatum.* It occurs mainly in immunocompromised individuals, more so in HIV-infected persons and usually with a CD4 counts<75 cells/μl. The fungus has been recovered most frequently from soil enriched with bird or bat droppings and is encountered in endemic forms in USA, Latin America, the Far East and Australia. It has two variants: *Histoplasma capsulatum var duboisii* found in Africa and *Histoplasma capsulatum var capsulatum* found in Latin America and other tropical countries. Lesions of the skin and bones predominate in the African form while pulmonary changes dominate the picture in the other form.[1] Although Panja and Sen first reported histoplasmosis from India in 1959, case reports of the disease have been few and far between.[2,3] In an Indian study from West Bengal, a prevalence of 9.4% positivity to histoplasmin sensitivity test has been reported.[4] Incidence is reported to be 2–5% in acquired immunodeficiency patients (AIDS) patients and <0.05% in non-HIV patients.[5]

Clinical manifestations of histoplasmosis are of three main types: pulmonary, progressive disseminated and chronic cavitatory forms.[6] On initial exposure to the fungus, the infection is self-limiting and restricted to lungs in 99% of the individuals while the remaining 1% progress to either disseminated or chronic disease involving the lungs, liver, spleen, lymph nodes, bone marrow and sometimes the skin and mucous membranes.[7]

Skin lesions may occur with all the three forms of histoplasmosis or, rarely, as primary cutaneous histoplasmosis. Cutaneous lesions occur in up to 17% of patients with disseminated histoplasmosis and can manifest as papules, pustules, plaques, ulcers, molluscum or wart-like lesions and, rarely, erythema nodosum.[8,9] Primary cutaneous histoplasmosis is very rare and can present with nodules, ulcers, abscesses or molluscum contagiosum-like lesions.[10] The route of infection is through direct inoculation of spores through skin and mucous membranes and thorn pricks are the most common mode of acquiring this variant of histoplasmosis. In our case, the splinter injury was the most likely portal of entry for the fungus. Primary cutaneous histoplasmosis in immunocompetent individuals itself is very rare, with hardly three case reports.[11] However, it is probably the first time in the literature that primary cutaneous histoplasmosis is being reported in a HIV-positive individual.

Diagnostic modalities include cultures, fungal stains of tissue or body fluids and tests for antigens and antibodies. Histoplasmin skin testing is not recommended for diagnostic purposes because of the high rate of positive reactions in endemic areas and variable duration of responses to the skin test. Skin biopsy can prove to be useful in establishing the diagnosis, especially in a set-up where facilities for serodiagnosis are not readily available. Antibody detection tests like complement fixation, DNA probes and radioimmunoassays are successful, but can be performed only in very few sophisticated centers. Detection of polysaccharide antigen in serum, urine or bronchoalveolar lavage in patients with disseminated histoplasmosis is a rapid and specific diagnostic method. Culture can be made from infective material such as blood, bone marrow or sputum, and they should be observed for 6–8 weeks before they may be considered negative. When biopsy is possible, sections of enlarged lymph nodes or tissues from splenic or hepatic puncture may show endothelial cells packed with histoplasma capsulatum.

Most acute forms of histoplasmosis in immunocompetent hosts resolve without specific treatment. Systemic antifungal treatment is indicated for severe acute pulmonary histoplasmosis, chronic pulmonary histoplasmosis, progressive disseminated histoplasmosis and any manifestation in an immunocompromised patient. The various drugs employed in treatment are amphotericin, ketoconazole, itraconazole and terbinafine, of which itraconazole is the drug of choice, except in severe systemic involvement, where amphotericin is preferred.[12] For disseminated fungal infections, suppressive therapy must be continued to prevent relapse. Even after clearing of lesions, therapy is to be continued for another 8 weeks at least. Relapse occurs in 10–20% of patients with disseminated infection and in as many as 80% of those with AIDS. Disseminated histoplasmosis in an immunocompromised host has a poor prognosis.

Our patient had many unique characteristics. Firstly, primary cutaneous histoplasmosis in immunocompromised individuals is itself extremely rare. The skin lesions mimicked photoallergic dermatitis and the presence of concurrent molluscum contagiosum lesions further added to the diagnostic dilemma. Another interesting fact was that the lesions initially responded to Fluconazole, later relapsed when treatment was stopped and finally improved with Itraconazole. The response of the lesions to oral antifungals in such a short duration along with the absence of any systemic symptoms, findings and investigations confirms the local nature of the disease manifestation. Such a condition in an immunosuppressed individual has probably not been reported in the literature earlier.

## CONCLUSION

Histoplasmosis is an opportunistic fungal infection occurring more commonly in immune-suppressed individuals. It has varied presentations, including pulmonary, progressive disseminated, chronic cavitatory and primary cutaneous forms. Primary cutaneous histoplasmosis is a very rare condition and we have hereby reported such a case in an immunosuppressed individual.
